# Factors associated with favourable outcome in large hemispheric infarctions

**DOI:** 10.1186/s12883-018-1148-7

**Published:** 2018-09-20

**Authors:** Jie Li, Ping Zhang, Simiao Wu, Xingyang Yi, Chun Wang, Ming Liu

**Affiliations:** 1 Department of Neurology, People’s Hospital of Deyang City, No.173, North Taishan Road, Deyang, 618000 Sichuan Province People’s Republic of China; 2 Stroke Clinical Research Unit, Department of Neurology, West China Hospital, Sichuan University, No. 37, GuoXue Xiang, Chengdu, 610041 Sichuan Province People’s Republic of China

**Keywords:** Large hemispheric infarction, Brain edema, Decompressive hemicraniectomy, Complication, Favorable outcome

## Abstract

**Background:**

Large hemispheric infarction (LHI) is a devastating condition with high mortality and poor functional outcome in most conservatively treated patients. The purpose of this study was to explore factors associated with favorable outcome in patients with LHI.

**Methods:**

We prospectively enrolled consecutive patients with LHI. Favorable outcome was defined as a modified Rankin Scale (mRS) score of 0 to 3 at 90 days. Multivariate logistic regression analysis was employed to identify the independent factors associated with favorable outcome.

**Results:**

Two hundred fifty-six cases with LHI were identified: 41 (16.0%) died during hospitalization, 94 (36.7%) died at 3 month, and 113 (44.1%) survived with favorable outcome at day 90. Compared with patients with unfavorable outcome, the favorable cases were younger (55.8 ± 14.7 vs. 66.2 ± 14.1), had less history of hypertension (38.9% vs. 59.3%), lower baseline NIHSS score (median NIHSS score 11 vs. 17), lower blood pressure on admission (systolic 134.7 ± 24.9 vs. 145.1 ± 26.1 mmHg; diastolic 80.2 ± 14.9 vs. 86.9 ± 16.2 mmHg; respectively), lower level of baseline serum glucose (7.2 ± 3.3 vs. 8.2 ± 3.3 mmol/L), a lower frequency of stroke-related complications (55.8% vs. 91.4%), more use of antiplatelets (93.8% vs. 57.1%) and statins (46.9% vs. 25.7%) in the acute phase of stroke, but less use of osmotic agents (69.9% vs. 89.3%), mechanical ventilation (1.8% vs. 20.0%) or decompressive hemicraniectomy (1.8% vs. 15.7%). Multivariable analysis identified the following factors associated with favorable outcome: age (odds ratio, OR 0.95, 95% confidence interval [CI] 0.92–0.98, *p* < 0.001), baseline NIHSS score (OR 0.90, 95% CI 0.84–0.96, *p* = 0.002), statins used in acute phase (OR 2.49, 95% CI 1.10–5.65, *p* = 0.029), brain edema (OR 0.05, 95% CI 0.01–0.21, *p* < 0.001) and pneumonia (OR 0.42, 95% CI 0.19–0.93, *p* = 0.032).

**Conclusion:**

More than one third of patients with LHI have relatively favorable clinical outcomes at 90 days. Younger age, lower baseline NIHSS score, absence of brain edema and pneumonia, and statins used in the acute phase were associated with favorable outcome of patients with LHI at 90 days.

## Background

Large hemispheric infarction (LHI), which usually refers to total or partial anterior circulation infarct caused by occlusion of internal carotid artery or the proximal middle cerebral artery (MCA), constitutes up to 10% of all supratentorial ischemic strokes [1]. Treatment of LHI has been a major unsolved problem in neurocritical care [2–4], with a high mortality rate of approximately 80% in conservatively treated patients [5]. Although decompressive hemicraniectomy (DHC) within 48 h after symptom onset has been proven to benefit highly selected patients with LHI [6], only 0.3% of all ischemic stroke patients would be eligible for DHC on the basis of the strict eligibility criteria in the European hemicraniectomy trials [7]. Until recently, there is a paucity of high quality evidence for doctors and family members to make decisions on treatment for patients with LHI.

The principal cause of death and poor functional outcome in patients with LHI is post-ischemic brain swelling, with subsequent raised intracranial pressure, neurological deterioration, midline shift and brain herniation [8]. Thus, prediction of malignant brain edema using clinical and radiological variables has been thoroughly investigated for the last two decades [9–13]. It is noted that not all patients with LHI would develop the malignant MCA infarction (mMCAi) that is characterized by severe edema and mass effect [14]. On the other hand, post- ischemic brain edema may produce a series of clinical manifestations from being clinically silent to rapid neurological deterioration or even death, depending on stroke location, infarct size, age of patient, and degree of premorbid atrophy [15]. Current available data for LHI in general emphasize the prediction and management of maligant brain edema, but offer limited discussion of the factors associated with favourable outcome in patients with LHI specifically. Therefore, we conducted a prospective cohort study that aimed to elucidate the factors that correlate with favorable outcome in patients with LHI.

## Methods

### Study design and subjects

Between Oct 1, 2011 and Sep 30, 2014, patients with either a first-ever stroke or recurrent stroke were registered consecutively after they were admitted to the Department of Neurology, People’s Hospital of Deyang City. Data were recorded at the time of assessment using a standardized structured form. Detailed methods for data collection have been previously described [16]. In present study, we enrolled patients who were admitted within 30 days from symptoms onset and diagnosed with LHI, which was defined as an ischemic stroke involving more than 50% of the territory of the MCA in computed tomography (CT) scan and/or standard magnetic resonance imaging (MRI), with or without involvement of the adjacent territories [17]. All patients had a brain CT scan before initial treatment. A second CT scan or MRI was performed within first 7 days of hospitalization. Other CT scans were performed if patients were suffered neurological deterioration, to identify brain edema or hemorrhagic transformation. We excluded cases with incomplete hospital records or missing imaging that would prevent complete data collection. We also excluded cases with preexisting score of more than 2 on the modified Rankin scale (mRS, a scale of 0 to 6, with 0 indicating no symptoms and 6 indicating death) and lived dependently [18].

### Data collection and outcome

Baseline data on age, sex, living environment (rural or urban), admission delay, initial stroke severity assessed by the National Institutes of Health Stroke Scale (NIHSS) score, baseline systolic and diastolic blood pressure, serum glucose on admission, vascular risk factors were collected. Vascular risk factors investigated in this study included hypertension, diabetes mellitus, dyslipidemia, coronary heart disease, atrial fibrillation, rheumatic heart disease, previous stroke/transient ischemic attack (TIA), current smoking and alcohol consumption, which have been described in a previous study [16]. The potential etiology of LHI was classified as large-artery atherosclerosis, cardio-embolism, stroke of other determined etiology and stroke of undetermined etiology according to the Trial of Org 10,172 in Acute Stroke Treatment (TOAST) criteria [19]. In-hospital treatment analyzed in our study included thrombolysis, DHC, mechanical ventilation, osmotic agents (such as mannitol), antiplatelet agents, anticoagulants, antihypertensives, statins and antidiabetics. DHC was conducted according the eligibility and exclusion criteria from the pooled analysis of three European randomized trials, when written informed consent was obtained from the patient or a legal representative [6]. Statins were used in patients whose stroke was presumed to be of atherosclerotic origin [20], or in patients regularly pretreated with statins [21], without consideration of baseline NIHSS score. Statins use was classified into two phases: previous stroke treatment or in the acute phase. Previous stroke treatment of statins referred to regular use of statins at least 1 month before stroke, and statins treatment in the acute phase was defined as statins initiated within 24 h after stroke onset and continuing for at least 3 days. The dosage was ≥20 mg per day for atorvastatin, ≥40 mg per day for simvastatin, or ≥ 10 mg per day for rosuvastatin. Stroke-related complications, including both neurological and medical complications during hospitalization, were reviewed by our staff members from hospital records after patient discharge. Neurological complications included brain edema, hemorrhagic transformation, seizures/epilepsy, central hyperthermia and recurrent stroke, while medical complications during hospitalization included pneumonia, urinary tract infection, gastrointestinal bleeding, electrolyte disturbance, urinary incontinence, acute renal failure, deep venous thrombosis, bedsore and falls [22].

Patients were followed up at 90 days after stroke onset by using questionnaires via telephone interview or letter inquiries. The Primary outcomes measures in our study were 3-month favorable outcome (defined as a mRS score of 0 to 3 [6]).

### Statistical analyses

Baseline characteristics were compared between favorable and unfavorable outcomes using student’s t-tests, Mann-Whitney U tests, χ^2^ tests or Fisher exact tests, as appropriate.Univariate analysis was performed to test variables which may affect outcome. The included variables were: (1) age group, (2) NIHSS score on admission, (3) all the risk factors surveyed in our study, (4) in-hospital treatments and (5) stroke related complication. The odds ratios (ORs) for variables associated with favorable outcomes were determined using multivariable logistic regression analyses by the forced entry method adjusted for variables with *p* < 0.05 on univariate analyses. All statistical levels quoted are 2-tailed and a value of *p* < 0.05 was considered significant for all results. The 95% confidence intervals (CI) were calculated to describe the precision of the estimates. All statistical analysis was performed with SPSS v21.0 (SPSS, Chicago, IL).

## Results

During the 3-year study period, 1542 patients with acute ischemic stroke were consecutively and prospectively registered. Of those, 256 (16.6%) patients with LHI were enrolled in the present study [mean age: 61.6 ± 15.3 years; 133 (52.0%) female; median NIHSS score on admission: 14]. Of these patients, 24 (9.4%) received DHC and 30 (11.7%) received mechanical ventilation. None of the patients received intra-arterial revascularization procedures. All LHI patients received cranial CT examination at least one time and 161 (62.9%) patients received MRI. The median length of stay in hospital (LOS) was 9.5 days (range, 1 to 78). 41 (16.0%) patients died during hospitalization. At 90 days, 3 (1.2%) patients was lost to follow-up. Among the entire cohort, 113 (44.1%) patients had favorable outcomes and 94 (36.7%) patients died at 90 days.

The baseline characteristics were compared between LHI patients with and without favorable outcomes (Table 1). Patients with favorable outcomes were younger (55.8 ± 14.7 vs. 66.2 ± 14.1, *p* < 0.001), had less history of hypertension (38.9% vs. 59.3%, *p* = 0.002), lower blood pressure on admission (systolic 134.7 ± 24.9 vs. 145.1 ± 26.1 mmHg, *p* = 0.001; diastolic 80.2 ± 14.9 vs. 86.9 ± 16.2 mmHg, *p* = 0.001; respectively), lower baseline serum glucose (7.2 ± 3.3 vs. 8.2 ± 3.3 mmol/L, *p* = 0.015), and a longer onset to admission time (26 vs. 24 h, *p* = 0.014) than those with unfavorable outcomes. The median total NIHSS score on admission was 11 in patients with favorable outcomes and 17 in those with unfavorable outcomes (*p* < 0.001). When stroke etiology of LHI is concerned, no significant difference was found between patients with and without favorable outcome (*p* = 0.222). The most common stroke subtype in both group was cardio-embolism (42.5% and 53.6%, respectively). 13 (11.5%) patients with favorable outcome and 13 (9.3%) without favorable outcome used statins previous to stroke. However, statins pretreatment did not have a significant association with favorable outcome in LHI in univariate analysis (*p* = 0.678). For in-hospital management of LHI, patients with favorable outcome more frequently used antiplatelets (93.8% vs. 57.1%, *p* < 0.001) and statins (46.9% vs. 25.7%, *p* < 0.001) in the acute phase of stroke, and less frequently used osmotic agents (69.9% vs. 89.3%, *p* < 0.001), mechanical ventilation (1.8% vs. 20.0%, *p* < 0.001), and DHC (1.8% vs. 15.7%, *p* < 0.001) (Table 2).

**Table 1:** Baseline characteristics of LHI patients with favorable and unfavorable outcome

	Unfavorable (mRS score 4–6) (*n* = 140)	Favorable (mRS score 0–3) (*n* = 113)	*P value*
Age(years)			
Mean ± SD	66.2 ± 14.1	55.8 ± 14.7	< 0.001*
Median(range)	69(17-99)	57(15–88)	< 0.001†
Female, n(%)	76(54.3)	56(49.6)	0.527‡
Rural population, n(%)	43(30.7)	41(36.3)	0.421‡
Time from onset(hours), median(range)	24(1–720)	26(3–720)	0.014†
NIHSS score on admission, median(range)	17(5–33)	11(4–31)	< 0.001†
SBP on admission (mm Hg)	145.1 ± 26.1	134.7 ± 24.9	0.001*
DBP on admission (mm Hg)	86.9 ± 16.2	80.2 ± 14.9	0.001*
Serum glucose on admission(mmol/L)	8.2 ± 3.3	7.2 ± 3.3	0.015*
Risk factors, n(%)			
Hypertension	83(59.3)	44(38.9)	0.002‡
Diabetes mellitus	32(22.9)	20(17.7)	0.350‡
Dyslipidemia	22(15.7)	25(22.1)	0.198‡
Coronary heart disease	21(15.0)	9(8.0)	0.117‡
Atrial fibrillation	69(49.3)	42(37.2)	0.057‡
Rheumatic heart disease	27(19.3)	31(27.4)	0.135‡
Current smoking	30(21.4)	27(23.9)	0.653‡
Alcohol consumption	23(16.4)	18(15.9)	0.915 ‡
Previous all strokes/TIA	27(19.3)	15(13.3)	0.236‡
Stroke in dominant hemisphere, n(%)	65(46.4)	60(53.1)	0.313‡
TOAST classification, n(%)			0.222‡
Large-artery atherosclerosis	27(19.3)	30(26.6)	
Cardioembolism	75(53.6)	48(42.5)	
Other determined etiology	9(6.4)	5(4.4)	
Undetermined etiology	29(20.7)	30(26.6)	
Statins pretreatment	13(9.3)	13(11.5)	0.678‡

Abbreviations: *SBP* systolic blood pressure, *DBP* diastolic blood pressure, *NIHSS* National Institutes of Health Stroke Scale, *GCS* Glasgow Coma Scale

*Student t test

†Mann–Whitney U test

‡χ2 test

**Table 2:** In-hospital Treatment of LHI patients with favorable and unfavorable outcome

	Unfavorable (mRS score 4–6) (*n* = 140)	Favorable (mRS score 0–3) (*n* = 113)	*P value*
In-hospital Treatments, n(%)			
Thrombolysis^a^	5(3.6)	2(1.8)	0.466
Decompressive surgery ^a^	22(15.7)	2(1.8)	< 0.001
Mechanical ventilation^a^	28(20.00)	2(1.8)	< 0.001
Osmotic agents^a^	125(89.3)	79(69.9)	< 0.001
Statins in acute phase ^a^	36(25.7)	53(46.9)	< 0.001
Antiplatelets ^a^	80(57.1)	106(93.8)	< 0.001
Anticoagulants^b^	6(4.3)	12(10.6)	0.083
Antihypertensives^b^	35(25.0)	25(22.1)	0.657
Antidiabetic drugs^b^	19(13.6)	16(14.2)	0.893

^a^Acute phase treatment

^b^Percentage is calculated for patients with indication of the treatment for secondary prevention

Patients with favorable outcome had less stroke-related complications (55.8% vs. 91.4%, *p* < 0.001; Table 3). Compared with patients with unfavourable outcomes, those with favorable outcomes had a significantly lower rate of malignant brain edema (4.4% vs. 53.6%, *p* < 0.001), pneumonia (36.3% vs. 67.1%, *p* < 0.001), gastrointestinal bleeding (3.5% vs. 17.9%, *p* = 0.001), electrolyte disturbance (19.5% vs. 40.0%, *p* < 0.001), acute renal failure (0 vs. 12.9%, *p* < 0.001), urinary incontinence (7.1 vs. 27.9%, *p* < 0.001) and bedsore (1.8 vs. 7.9%, *p* = 0.042). However, there was no significant difference in the events rate of hemorrhagic transformation, seizures/epilepsy, central hyperthermia and recurrent stroke between patients with and without favorable outcomes (all *p* > 0.05).

**Table 3:** Stroke-related complication during hospitalization of LHI patients with favorable and unfavorable outcome

	Unfavorable (mRS score 4–6) (*n* = 140)	Favorable (mRS score 0–3) (*n* = 113)	*P value*
Stroke-related complications, n(%)	128(91.4)	63(55.8)	<0.001
Neurological complications, n(%)			
Brain edema	75(53.6)	5(4.4)	<0.001
Hemorrhagic transformation	46(32.9)	25(22.1)	0.068
Seizures/epilepsy	11(7.9)	7(6.2)	0.634
Central hyperthermia	9(6.4)	2(1.8)	0.118
Recurrent stroke	3(2.1)	0(0)	0.256 *
Medical complications, n(%)			
Pneumonia	94(67.1)	41(36.3)	<0.001
Urinary tract infection	14(10.0)	5(4.4)	0.148
Gastrointestinal bleeding	25(17.9)	4(3.5)	0.001
Electrolyte disturbance	56(40.0)	22(19.5)	<0.001
Acute renal failure	18(12.9)	0(0)	<0.001*
Urinary incontinence	39(27.9)	8(7.1)	<0.001
Bedsore	11(7.9)	2(1.8)	0.042
Deep venous thrombosis	6(4.3)	3(2.7)	0.735
Falls	3(2.1)	0(0)	0.256*

^*^Fisher exact test

Variables that had potential confounding effects on the 3-month favorable outcome in univariate analysis (*p* < 0.05) and the final results of multivariate logistic regression are shown in Table 4 with OR and 95% CI. After adjusting for potential confounding factors excluding stroke-related complications (see model 1), age (OR 0.95, 95% CI 0.92–0.97), baseline NIHSS score (OR 0.87, 95% CI 0.82–0.92), history of hypertension (OR 0.38; 95% CI 0.18–0.78), DHC (OR 0.11; 95% CI 0.01–0.92), statins use in acute phase (OR 2.75, 95% CI 1.35–5.59) were independently associated with 3-month favorable outcome in LHI patients(all *P* < 0.05). When stroke-related complications were included into the multivariate logistic regression (see model 2), history of hypertension and DHC were no longer independently associated with 3-month favorable outcome, however, brain edema (OR 0.05, 95% CI 0.01–0.21) and pneumonia (OR 0.42, 95% CI 0.19–0.93) were independently associated with 3-month favorable outcome in LHI patients in addition to age (OR 0.95, 95% CI 0.92–0.98), baseline NIHSS score (OR 0.90, 95% CI 0.84–0.96) and statins used in acute phase(OR 2.49, 95% CI 1.10–5.65) (all *P* < 0.05).

**Table 4:** Factors associated with 3-month favorable outcome in LHI patients

Varibles	Univariate analysis	Multivariate analysis (model 1)^*^	Multivariate analysis (model 2)^*^
Age	0.95(0.93–0.97)	0.95(0.92–0.97)	0.95(0.92–0.98)
Baseline NIHSS score	0.86(0.82–0.90)	0.87(0.82–0.92)	0.90(0.84–0.96)
Hypertension	0.44(0.26–0.73)	0.38(0.18–0.78)	
Baseline serum glucose	0.90(0.82–0.98)		
Decompressive surgery	0.10(0.02–0.42)	0.11(0.01–0.92)	
Ventilatory Support	0.07(0.02–0.31)		
Antiplatelets	5.24(2.23–12.31)		
Statins	2.55(1.50–4.33)	2.75(1.35–5.59)	2.49(1.10–5.65)
Pneumonia	0.28(0.17–0.47)		0.42(0.19–0.93)
Gastrointestinal bleeding	0.17(0.06–0.50)		
Electrolyte disturbance	0.36(0.20–0.65)		
Bedsore	0.21(0.05–0.97)		
Urinary incontinence	0.20(0.09–0.44)		
Brain edema	0.04(0.02–0.10)		0.05(0.01–0.21)

Variables which had a significant association with favorable outcome in univariate analysis were listed (*P*<0.05). Figures in parentheses are 95% confidence intervals (CI)

Model 1: adjusted for variables with *p* < 0.05 on univariate analyses excluding stroke-related complication

Model 2: adjusted for variables with *p* < 0.05 on univariate analyses including stroke-related complication

^*^Adjusted odds ratios (OR) with *p* < 0.05 in the multivariate logistic regression analysis

## Discussion

It has been reported that advanced age, initial stroke severity (NIHSS or Glasgow coma scale score), involvement of the adjacent (anterior or posterior cerebral artery) territories, anisocoria, early neurological deterioration, coronary artery disease, and internal carotid artery occlusion were the predictors of mortality and poor functional outcome in patients with LHI. However, all of these studies mentioned above were conducted in DHC cohorts [17, 23–29]. A number of clinical and radiological predictors of malignant brain edema, such as younger age, female sex, no previous ischemic stroke, history of hypertension, dominant hemisphere, have been extensively investigated for the last two decades [30]. A meta analysis included 23 studies and found that large infarct size especially involving more than 50% of the MCA territory and a perfusion deficit of more than 66% on CT scan to be the most reliable predictors of maligant edema formation [13]. Nevertheless, not all patients with LHI would develop the mMCAi [14]. Thus, we conducted a prospective, non-selective cohort of patients with LHI to elucidate the factors that directly correlate with favorable outcome at 90 days. In the present study, we found that more than one third of patients with LHI have relatively favorable clinical outcomes at 90 days. In the multivariable logistic regression analyses, adjusted for potential confounding factors, we found that (1) younger age, lower baseline NIHSS score and statins used in the acute phase were independently related to favorable outcomes; (2) It was maligant brain edema and increased risk of pneumonia concommitant with surgery, rather than decompressive surgery itself independently associated with unfavorable outcome.

### Age

Whereas age is the leading risk factor and the strongest predictor of clinical outcome after ischemic stroke [31, 32], the influence of age on outcome has not yet been well investigated in LHI. As a general rule, young and middle-aged patients have less compensation capacity for space-occupying intracranial lesions than older patients with cerebral atrophy, however, those patients tend to have fewer comorbid conditions that are likely to increase the risk of mortality and poor functional outcome [33]. The result of our study is in line with previous studies conducted in LHI patients that received DHC [23–25], which reported that younger age was associated with favorable outcome in non-selective patients with LHI.

### Statins in acute phase

Of note, in our cohort, among those patients with favorable outcome at 90 days, 46.9% (53/113) use statins in acute phase of stroke, but only 25.7% (36/140) of those without favorable outcome use statins. Results from multivariable analysis of our cohort showed that statins use in acute phase was an independent predictor for 3-month favorable outcome in patients with LHI, regardless of the models adjusting for potential confounders by including or excluding stroke-related complications. Statins have been widely used for primary and secondary prevention of stroke for many years. Recently, statins have been found to play an important role in the neuroprotection of acute ischemic stroke in animal models, due to its pleiotropic effects besides lipid-lowering such as vasodilatory, angiogenesis, neurogenesis, synaptogenesis, antithrombotic, anti-inflammatory, antioxidant and anticonvulsant effects [34–36]. Meanwhile, cerebral arterial occlusion has been associated with reduced infarct volume and improved neurological function with use of statins in experimental animal models [34–36]. A recent research showed that pretreatment with statins is associated with better outcomes regarding neurological improvement, disability, survival, and stroke recurrence in large artery atherosclerotic stroke [37]. A meta-analysis including 113,148 stroke patients suggested an association between prestroke statins use and improved stroke outcome at 90 days [38]. Based on available evidence, it is recommended to continue with prestroke statin treatment in the acute phase [21]; however, routinely prescribing statins as neuroprotective agents is still controversial. Moreover, there are limited available data evaluating the association between statins use in acute phase and favorable outcome in patients with LHI. The result of our study provides initial evidence that statins used in acute phase may be associated with favorable outcome in patients with LHI. Considering the limitations of observational study, randomized controlled trials are needed to validate the findings.

### DHC and stroke-related complications

In the present study, we found that patients with favorable outcome less frequently had received DHC and mechanical ventilation than those without favorable outcomes. Multivariable analysis not including stroke-related complications showed that DHC was independently associated with unfavorable outcome (model 1). However, when stroke-related complication was taken into consideration, DHC was no longer an independent factor correlated with unfavorable outcome, while brain edema and pneumonia were independently associated with unfavorable outcome (model 2). Data from a previous pooled analysis of three randomized trials had demonstrated that DHC reduced mortality without increasing the risk of very severe disability among patients 60 years of age or younger with mMCAi [6]. DESTINY II trial indicated that DHC increased survival among patients older than 60 years of age with mMCAi, but most survivors were left with disabilities and needed assistance for daily living [39], which was also confirmed in a small sample randomized trial with Chinese patients [40]. The results of present study showed that DHC was no longer independent factor associated with unfavorable outcome in patients with LHI after adjusting for age, baseline NIHSS score and stroke-related complication. Therefore, we could reasonablely speculate that it was malignant brain edema and increased risk of pneumonia concomitant with surgery, rather than DHC itself were independently associated with unfavorable outcome. In-hospital medical complications due to DHC can impact on the clinical outcomes of the patient with LHI. A national inpatient sample database over a 6-year period in the United States found the rates of pneumonia to be 11.1% in 252 patients studied and was associated with an increased rate of mortality [41]. In a cohort study with patients undergoing DHC for space-occupying LHI, 42.4% of the patients suffered from at least one postoperative complication, with pulmonary problems being the most common cause (39% of all complications) [17]. Stroke-related pneumonia was the most common medical complication in our study (36.3% in patients with favorable outcomes and 67.1% in those with unfavorable outcomes) and patients complicated with pneumonia showed 42% (95% CI 0.19–0.93) chance of favorable outcome compared with those patients without, after adjusting for age, baseline NIHSS score and other confounders. Since most pneumonia are potentially preventable or treatable, doctors should pay rigorous attention to the prevention, early detection and treatment of stroke-related pneumonia because of the higher events risk and concomitant poor outcome.

### Limitations

The results of the present study should be interpreted with caution given its limitations. First, it was a single hospital-based study, with limited generalisability. Some patients with LHI might not be hospitalized, especially those who died before admitted to hospital, so we could not exclude inclusion bias in this study. Second, we only conduct a 3-month follow-up so that the predictive effect on long-term outcomes remains unclear. Therefore, we cannot advise whether the factors we identified associated with favorable outcome in patients with LHI also have a long-term effect. Third, since our hospital is one of national advanced stroke center in China, admission to a stroke unit, early mobilisation, bedside swallow evaluation and rehabilitation have become routine practice in even every stroke patients, our prognostic model of LHI did not incorporate these factors such as admission to a stroke unit, early mobilisation, swallow status or other life-threatening impairment. Finally, follow-up in our study was performed by telephone interview or postal questionnaire instead of a clinic visit which may result in a reporting bias.

## Conclusions

We identified that more than one third of patients with LHI have relatively favorable clinical outcomes at 90 days.Younger age, lower baseline NIHSS score, absence of brain edema and pneumonia and statins used in the acute phase may be directly related to favorable outcome in patients with LHI. The result of our study provides preliminary evidence that the use of statins in the acute phase was associated with favorable outcome in patients with LHI. Future studies are needed to verify the findings of current study.

## Abbreviations

95%CI: 95% confidence interval
CT: Computed tomography
DHC: Decompressive hemicraniectomy
LHI: Large hemispheric infarction
MCA: Middle cerebral artery
mMCAi: Malignant middle cerebral artery infarction
MRI: Magnetic resonance imaging
mRS: Modified Rankin Scale
NIHSS: National Institutes of Health Stroke Scale
OR: Odds ratio
